# Trends in Age‐Adjusted Cardiovascular Mortality Rates Among the Diabetic Population of the United States: 1999–2020, CDC WONDER Retrospective Study

**DOI:** 10.1002/hsr2.72421

**Published:** 2026-05-10

**Authors:** Aiman Baloch, Quang Dai La, David M. Lo, Jasneel Kahlam

**Affiliations:** ^1^ Mekran Medical College Turbat Balochistan Pakistan; ^2^ Department of Biology Texas A&M University College Station Texas USA; ^3^ Rutgers University New Brunswick New Jersey USA; ^4^ Department of Family Medicine Garnet Health Medical Center Orange County New York USA; ^5^ Department of Internal Medicine Stony Brook Southampton Hospital Southampton New York USA

**Keywords:** cardiovascular diseases, CDC WONDER data analysis, diabetes mellitus, mortality trends, racial CVD trends

## Abstract

**Background and Aims:**

This study examines trends in cardiovascular disease (CVD)‐related mortality among individuals with diabetes mellitus (DM) in the United States from 1999 to 2020, focusing on age‐adjusted mortality rates (AAMRs) across demographic and geographic subgroups.

**Methods:**

Using the CDC WONDER database, we analyzed death certificate data and calculated AAMRs standardized to the 2000 US population. Joinpoint regression was used to analyze annual percentage changes (APCs) in AAMRs by sex, race/ethnicity, and geographic region. Statistical significance was determined at *p* < 0.05.

**Results:**

From 1999 to 2020, 1,854,384 deaths were attributed to circulatory disorders and DM, with an average AAMR of 25.1%. AAMRs showed a steady decline from 1999 (31.3/100,000) to 2014 (21.9/100,000), followed by an increase to 25.6/100,000 in 2020. Gender analysis revealed significant declines in AAMRs for both males and females, though the rate of decline slowed after 2012. Racial disparities were evident: non‐Hispanic Whites and Asian or Pacific Islanders experienced reversals in mortality trends after earlier declines, while Hispanic or Latino groups exhibited a steep rise in AAMRs from 2016 to 2020 (APC = 10.84%). Geographic analysis highlighted considerable variation in mortality rates across states, with Oklahoma reporting the highest AAMR (59.3/100,000).

**Conclusions:**

While overall CVD mortality in individuals with DM has improved, recent increases in AAMRs and persistent demographic and geographic disparities underscore the need for targeted public health strategies. Addressing these disparities is critical to sustaining progress and improving outcomes for vulnerable populations.

## 1 Introduction

In 2021, approximately 529 million people lived with diabetes worldwide, resulting in a global age‐standardized diabetes prevalence of 6.1% [1]. Diabetes remains a significant public health concern, as its prevalence continues to rise in both developed and developing countries. This increase places substantial pressure on healthcare systems globally as they contend with the burden of managing diabetes and its associated complications [2]. According to the CDC, approximately 37.3 million people, or 11.3% of the US population, had diabetes, whether diagnosed or undiagnosed, in 2019 [3]. This included 37.1 million adults aged 18 or older, representing 14.7% of all US adults [3]. This growing prevalence highlights the need for effective prevention and management strategies to mitigate the health and economic impacts of diabetes [4]. Cardiovascular diseases (CVD) are the leading cause of illness and death among individuals with type 1 and type 2 diabetes mellitus (DM) [5, 6, 7]. Despite advances in medical treatments and preventive measures, the burden of CVD in diabetic populations remains disproportionately high [8].

Both CVD and diabetes share similar risk factors, such as age, family history, obesity, physical inactivity, unhealthy diet, high blood pressure, high cholesterol, and smoking, and are linked through common pathways [9]. These overlapping risk factors create a synergistic effect that amplifies the morbidity and mortality associated with each condition. Research continues to explore how targeted interventions addressing these shared pathways could significantly reduce the disease burden in affected populations by improving prevention, early detection, and management of CVD in these at‐risk groups [10]. Understanding these connections is critical to developing holistic approaches to patient care that address the underlying mechanisms of both diseases [11].

Our analysis investigates trends in CVD‐related mortality among the diabetic population in the United States, emphasizing key demographic characteristics such as age, gender, and race, and understanding how they may influence health trends. Furthermore, we examined state‐wise age‐adjusted mortality rates (AAMRs). Our findings seek to assess demographic and regional disparities in CVD mortality in diabetic patients, which can potentially improve outcomes [3].

## 2 Methods

### 2.1 Study Setting and Population

We analyzed data from the CDC WONDER database covering January 1, 1999 to December 31, 2020. This analysis focused on death certificates from the Multiple Cause‐of‐Death Public Use records for participants with circulatory disorders and DM. The International Statistical Classification of Diseases, Tenth Revision, Clinical Modification (ICD‐10) includes codes E10–E14 for various types of DM. These codes were used to identify cases of diabetes among the US population in the multiple causes of death section from 1999 to 2020. ICD codes for the cardiovascular system were determined using I00–I99 to measure cardiovascular‐related mortality in the diabetic population as a sensitivity analysis on underlying causes of death. This research study is exempt from local institutional review board approval as it employs a public‐use data set sourced from a government entity.

### 2.2 Data Abstraction

To evaluate age‐adjusted CVD mortality across various subtypes of DM, we collected data on population sizes, years, demographics, race, and geographic regions. Race was categorized as reported on death certificates, following standards set by the United States (US) Office of Management and Budget. The racial classifications included non‐Hispanic (NH) White, NH Black or African American, Hispanic or Latino, NH American Indian or Alaskan Native, and NH Asian. Geographic regions were defined as US states.

### 2.3 Statistical Analysis

To analyze national trends in cardiovascular mortality rates among the diabetic population, we calculated AAMRs/100,000 individuals from 1999 to 2020. These rates were organized by year, sex, race, state, and region, and we included 95% confidence intervals (CIs) in our calculations. Crude mortality rates were determined by dividing the number of cardiovascular‐related deaths by the corresponding US population for each year.

We calculated AAMRs by standardizing deaths to the 2000 US population. To assess the national annual trends in CVD‐related mortality in the DM population, we used the Joinpoint Regression Program (Joinpoint V 5.3.0, National Cancer Institute).

We used this tool to determine the annual percent change (APC) in AAMRs and 95% CIs. This method identifies significant changes in AAMR over time by fitting log‐linear regression models to capture temporal variations. APCs and their 95% CIs for AAMR were calculated for the identified segments of the line connecting the joinpoints, utilizing the Weighted Bayesian Information Criterion (BIC) test.

APCs were classified as increasing or decreasing if the slope representing the change in mortality significantly differed from zero, as assessed by two‐tailed *t*‐testing. A *p* value of < 0.05 was considered significant.

## 3 Results

### 3.1 Overall Mortality Trends

The AAMR and death trends from 1999 to 2020 are displayed in the data. Those with circulatory illnesses and DM accounted for 1,854,384 deaths, and the overall average age‐adjusted mortality was 25.1%, as shown in Table 1.

**Table 1:** Yearly mortality metrics: total death counts and age‐adjusted mortality rate (AAMR) trends from 1999 to 2020, reflecting temporal changes in mortality.

Year	Deaths	Age‐adjusted rate	Year	Deaths	Age‐adjusted rate
1999	85,547	31.3	2010	79,041	23.8
2000	84,910	30.7	2011	78,776	23.1
2001	85,144	30.3	2012	79,598	22.7
2002	85,539	30	2013	81,357	22.7
2003	85,077	29.3	2014	80,205	21.9
2004	82,996	28.1	2015	82,365	21.9
2005	83,830	27.9	2016	84,764	22.1
2006	80,927	26.3	2017	88,377	22.5
2007	80,185	25.6	2018	89,211	22.2
2008	80,320	25.1	2019	90,804	22.2
2009	78,795	24.2	2020	106,616	25.6

### 3.2 Annual Trends in Mortality

**Figure 1:**
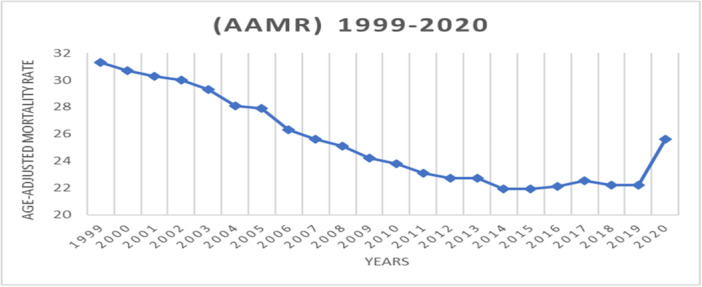
Trend of age‐adjusted mortality rates (AAMRs) in the United States from 1999 to 2020, showing overall temporal changes in mortality standardized to the 2000 US population.

Over the years, the overall death toll varied, first dropping from 85,547 in 1999 to 78,795 in 2009. But after 2014, there was a discernible rise, reaching a high of 106,616 fatalities in 2020. Additionally, from 31.3 per 100,000 in 1999 to a low of 21.9 in 2014 and 2015, the AAMR steadily declined. However, this trend reversed, with the rate increasing to 25.6 in 2020, as illustrated in Figure 1.

### 3.3 Annual Trends by Gender

**Figure 2:**
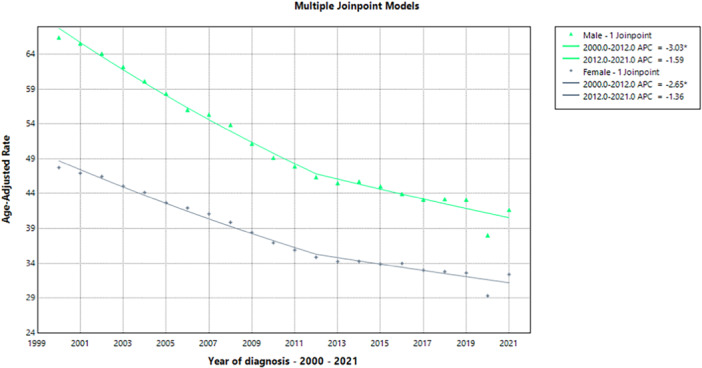
Gender‐specific trends in age‐adjusted regression analysis of mortality rates, illustrating differences in temporal patterns between male and female populations across the study period.

A Joinpoint regression model for the years 1999–2020 was used to assess the trends in the age‐adjusted rates for both males and females. Details of the model estimates, estimated joinpoints, model selection methods, and tests for the number of joinpoints are provided in Tables S1–S4. Two noteworthy patterns emerged from the combined cohort's APC: The APC showed a substantial drop from 2000 to 2012 (−2.87%, 95% CI: −3.37 to −2.40, *p* < 0.0001) and a slower but still significant decline from 2012 to 2021 (−1.50%, 95% CI: −1.92 to −1.06, *p* < 0.0001). The combined cohort's Average Annual Percent Change (AAPC) for the entire period (1999–2020) was −2.28% (95% CI: −2.51 to −2.05, *p* < 0.0001), as demonstrated in Figure 2, Tables 2 and 3.

**Table 2:** Annual percent change (APC) trends showing year‐to‐year variation in age‐adjusted mortality rates (AAMR) between males and females.

Cohort	Segment	Lower endpoint	Upper endpoint	APC	Lower CI	Upper CI	Test statistic (*t*)	Prob > |t|
Male and female	1	2000	2012	−2.7884*	−3.1766	−2.3987	−14.9131	< 0.000001
Male and female	2	2012	2021	−1.4439*	−2.0678	−0.8160	−4.8318	0.000156

*Note:* * means *p*‐value ≤ 0.05.

**Table 3:** Average annual percent change (AAPC) demonstrating the overall long‐term trends in AAMR for males and females across the entire study period.

Cohort	Range	Lower endpoint	Upper endpoint	AAPC	Lower CI	Upper CI	Test statistic (t)	Prob > |t|
Male and female	Full range	2000	2021	−2.2144*	−2.5368	−1.8910	−13.2917	< 0.000001

*Note:* * means *p*‐value ≤ 0.05.

### 3.4 Racial Disparities in Mortality Rates

**Figure 3:**
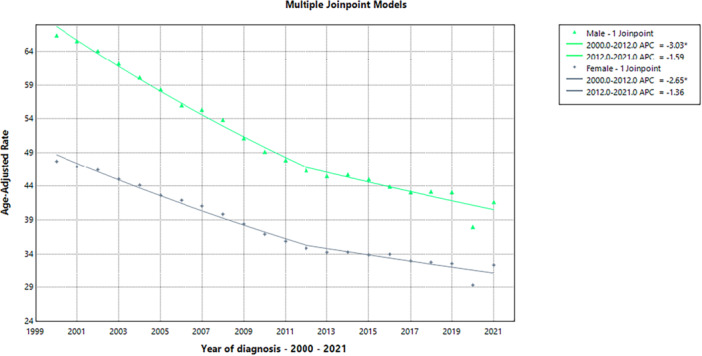
Graphical representation of trends in age‐adjusted mortality rates (AAMRs) by race and ethnicity in the United States, comparing Hispanic, non‐Hispanic White, Asian or Pacific Islander, Black or African American, and American Indian or Alaska Native populations over the study period.

Using Joinpoint regression models, the trends in age‐adjusted rates for various racial/ethnic groupings between 1999 and 2020 were assessed. From 1999 to 2020, different racial and ethnic groupings showed different APC trends, according to the statistical study. For analyses stratified by race/ethnicity, the model estimates, estimated joinpoints, model selection criteria, and tests for the number of joinpoints are detailed in Tables S5–S8. There were two notable periods for non‐Hispanic Whites: the APC experienced a substantial decline of −2.58% from 1999 to 2012, a nonsignificant increase of 0.36% from 2012 to 2018, and a significant increase of 6.33% from 2018 to 2020. For non‐Hispanic Asian or Pacific Islanders, there was a major loss of −3.65% from 1999 to 2013, followed by a nonsignificant decrease of −0.48%. Between 1999 and 2018, non‐Hispanic Blacks showed a consistent decrease of −2.74% (significant), which was followed by a significant increase of 12.44% between 2018 and 2020. Between 2011 and 2020, there was a nonsignificant change of 0.043%, following a substantial fall of −2.94% among non‐Hispanic American Indians or Alaska Natives until 2011. Last but not least, there was a notable decline of −3.09% among Hispanic or Latino people between 1999 and 2016, followed by a notable rise of 10.84% between 2016 and 2020. These trends show how several demographic groups have changed during the past 20 years, as shown in Figure 3 and Table 4.

**Table 4:** Racial and ethnic variations in annual percent change (APC) trends in age‐adjusted mortality rates.

Cohort	Segment	Lower endpoint	Upper endpoint	APC	Lower CI	Upper CI	Test statistic	*p*
White – 2 joinpoints	1	1999	2012	−2.5828*	−3.4853	−2.1892	—	0.014797
White – 2 joinpoints	2	2012	2018	−0.3561	−2.6338	1.3562	—	0.389122
White – 2 joinpoints	3	2018	2020	6.3307*	1.3681	9.0587	—	0.002799
Asian or Pacific Islander – 2 joinpoints	1	1999	2013	−3.6454	−5.9588	0.4098	—	0.055989
Asian or Pacific Islander – 2 joinpoints	2	2013	2018	−0.4813	−4.1394	1.6940	—	0.371526
Asian or Pacific Islander – 2 joinpoints	3	2018	2020	7.5642*	1.8085	11.2332	—	0.001200
Black or African American – 1 joinpoint	1	1999	2018	−2.7433*	−3.2542	−2.3429	—	0.000800
Black or African American – 1 joinpoint	2	2018	2020	12.4379*	3.0777	16.8677	—	0.011198
American Indian or Alaska Native – 1 joinpoint	1	1999	2011	−2.9426*	−4.0428	−2.2457	—	< 0.000001
American Indian or Alaska Native – 1 joinpoint	2	2011	2020	0.4256	−0.4226	1.9031	—	0.295141
Hispanic or Latino – 1 joinpoint	1	1999	2016	−3.0916*	−8.7645	−1.1827	—	0.020396
Hispanic or Latino – 1 joinpoint	2	2016	2020	10.8380*	0.5528	33.2579	—	0.037193

*Note:* * means *p*‐value ≤ 0.05.

### 3.5 Geographic Disparity in Mortality Rates

**Figure 4:**
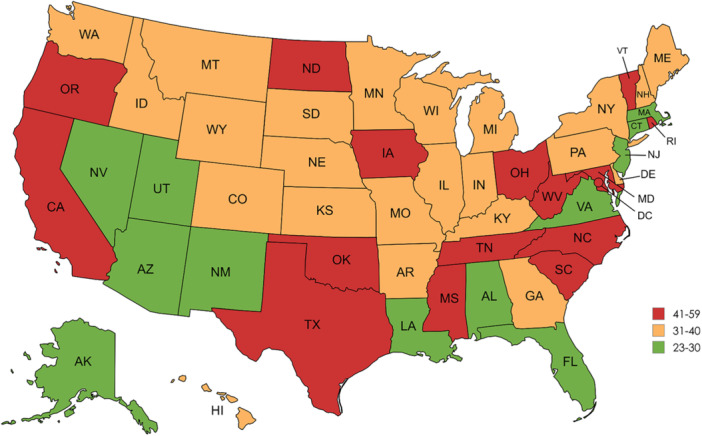
Geographic variation in age‐adjusted mortality rates (AAMRs) across US states: a state‐level visualization of mortality disparities.

There is notable variation in the age‐adjusted death rates across states, as summarized in Table 5. With 59.3 deaths/100,000, Oklahoma has the highest mortality rate, followed by Vermont (55.9) and Mississippi (53.5). The states with the lowest mortality rates, however, are Nevada (28.9), Utah (27.7), and Connecticut (27.5). State‐wise trends in AAMR ranges are further visualized in Figure 4.

**Table 5:** State‐wise age‐adjusted mortality rates (AAMRs) revealing geographic health disparities in the United States.

States	Age‐adjusted mortality rate	States	Age‐adjusted mortality rate
Oklahoma	59.3	Minnesota	36.8
Vermont	55.9	Wyoming	36.7
Mississippi	53.5	Missouri	35.9
District of Columbia	53.4	Idaho	35.7
West Virginia	50.6	New Hampshire	35.1
Ohio	49.4	Arkansas	34.5
California	46.6	New York	34.3
Texas	44.1	Colorado	34.1
Tennessee	44	Illinois	32.9
Rhode Island	43.6	Montana	32.7
North Carolina	43.3	Georgia	32.3
Oregon	42.8	Kansas	31.8
Iowa	41.7	Maine	31.6
Maryland	41.6	Alabama	30.1
North Dakota	41.6	Virginia	30
South Carolina	41	Louisiana	30
Kentucky	40.4	New Mexico	29.7
Washington	39.3	Alaska	29.3
Michigan	39.2	New Jersey	29
Wisconsin	38.9	Nevada	28.9
Nebraska	38.9	Utah	27.7
South Dakota	38.8	Connecticut	27.5
Indiana	38.4	Arizona	25.6
Hawaii	37.9	Massachusetts	24.5
Pennsylvania	37.6	Florida	23.4
Delaware	37.1		

## 4 Discussion

Significant improvements in healthcare, public health campaigns, and the removal of key risk factors for preventable diseases, such as smoking, may have contributed to the decline in the AAMR observed between 1999 and 2014 [12]. This time frame corresponds with the wider use of interventions, including enhanced access to preventive care, better medical treatments, and increased health education. Nonetheless, the upward trend in AAMR, which began in 2016 and spiked in 2020, raises concerns about new or worsening contributors to diabetes and CVD mortality. Emerging public health challenges, inequalities in healthcare access, and modifications in disease occurrence and treatment are possible explanations for this development [13, 14]. These findings emphasize the need to reevaluate current policies and prioritize interventions.

Although age‐adjusted mortality declined during the early study period, total deaths increased in later years. This may suggest that a greater number of individuals are affected by these conditions, potentially reflecting population aging and an increasing prevalence of diabetes and CVD. These relationships underscore the importance of addressing broader population‐level health factors while continuing to reduce individual risk. Our findings are consistent with previous studies published in *JAMA Cardiology* and *Diabetes Care*, which reported slowing declines and plateauing trends in cardiometabolic mortality. Similarly, those studies observed elevated AAMRs associated with diabetes and CVD characterized by largely nonsignificant APCs [15, 16]. In contrast, our analysis revealed pronounced subgroup differences, particularly among racial and ethnic populations.

Racial and ethnic disparities were particularly evident. After 2018, upward trends were observed in Asian or Pacific Islander and White populations, suggesting potential emerging health challenges or disparities in care. In contrast, mortality rates for American Indian or Alaska Native populations stabilized following 2011–2016, which may indicate stalled progress and highlight the need for renewed targeted interventions [17, 18]. Between 2016 and 2020, death rates among Hispanic/Latino populations increased, with an APC of 10.84%. This trend reflects urgent health challenges, including limited healthcare access, language barriers, social vulnerability, and the effects of public health crises. Addressing underlying contributors (such as socioeconomic inequality, insufficient healthcare access, and high disease burden) may benefit from targeted, equity‐focused public health strategies and inclusive healthcare policies [19].

Importantly, the significant uptrend in AAMR among African American individuals from 2018 to 2020 is particularly concerning. Cardiovascular mortality rates have remained higher among African American adults than among any other racial or ethnic group in the United States over the past several decades. A temporal analysis of cardiovascular deaths in the United States demonstrated that from 1999 to 2020, the age‐adjusted rate of cardiovascular death was higher for African American individuals than for any other racial group, indicating persistent disparities in mortality due to CVD [19, 20]. Consistent with our findings of rising AAMRs in recent years, the sharp increase observed in this population may reflect a continuation or worsening of longstanding socioeconomic inequities. Furthermore, adults with diabetes have consistently demonstrated significant health disparities based on income level, race/ethnicity, and healthcare access, with lower income‐to‐poverty ratios and food insecurity associated with higher prevalence of myocardial infarction, stroke, and heart failure [21].

One contributing factor to the recent reversal of mortality trends may be the Coronavirus (COVID‐19) pandemic. A CDC WONDER analysis found that although diabetes‐related CVD mortality declined by nearly 29% from 1999 to 2019, the first year of the COVID‐19 pandemic was associated with a 15.5% increase in age‐adjusted diabetes‐related cardiovascular mortality, contributing an estimated 16,009 excess deaths in 2020 relative to prior trends [22]. These findings suggest that pandemic‐related disruptions, including reduced healthcare access and interruptions in chronic disease management, may have contributed to the observed spike. Furthermore, national population data indicate that overall cardiovascular mortality in the United States had already experienced slowing declines after 2010, with near‐flat or increasing trends across multiple states and counties [23].

Marked interstate disparities in cardiovascular mortality among individuals with diabetes likely reflect structural factors such as rurality, healthcare access, and Medicaid availability. Rural states such as Mississippi and Oklahoma face higher diabetes prevalence and mortality, partly due to healthcare provider shortages and limited health infrastructure [24]. Additionally, states that expanded Medicaid experienced significantly smaller increases in cardiovascular mortality—approximately 4 fewer deaths per 100,000 per year—compared to nonexpansion states [24]. Delayed policy implementation and rural healthcare limitations may therefore contribute to the geographic variation observed in this study.

Overall, these findings demonstrate the complex interaction between biological, social, and structural determinants in shaping cardiovascular mortality among individuals with diabetes. Continued efforts to address inequities, strengthen healthcare systems, and implement adaptable public health strategies will be necessary to reduce the overall disease burden in high‐risk populations.

## 5 Limitation

The study has several limitations that need to be acknowledged. First, the absence of longitudinal patient‐level data prevents tracking the progression of cardiovascular risk among the diabetic population over time and understanding the impact of comorbidities. Also, a key limitation of this study is the unavailability of age‐stratified and diabetes type–specific data, which may mask important variations across different subpopulations. Additionally, the data set does not include critical risk factors, such as smoking, diet, obesity, physical activity, or medication use, which are health determinants that could serve as confounding variables. Lastly, the lack of information on healthcare access, including insurance status and the availability of specialized care, limits the ability to assess disparities in outcomes and other socioeconomic indicators. Addressing these gaps in future research could strengthen the findings and offer deeper insights into CVD mortality among individuals with diabetes.

In addition to the limitations previously described, the use of death certificate data is subject to the bias of misclassification that can impact the trends and differences among subgroups. Death certificates lack sensitivity for chronic diseases such as CVD and diabetes. In one US study, only 6.2% of those who died with a known diagnosis of diabetes had it listed on their death certificate. Of overall cases of diabetes, the sensitivity of diabetes reported on death certificates has been documented to be limited (35%–54%), while the specificity of death certificates has been found to be very high [25]. Death certificates may also contain errors in the assignment of cause of death (the degree to which the cause is listed as contributing to the actual cause) which has been found to significantly alter mortality statistics. In validation analysis, more than half of the death certificates in a validation study were found to have at least one major error that impaired the assignment of an ICD‐10 code, which could lead to an under/over estimation of cause‐specific mortality and may be particularly problematic for those with many comorbidities [26]. The lack of individual‐level data on risk factors (smoking, control of hypertension, medications, socioeconomic status) in the CDC WONDER makes it impossible to control for these key determinants of causality in the interpretation of observed disparities between groups, therefore caution is recommended in making causal inference on observed disparities until individual data can be analyzed.

## 6 Conclusion

Between 1999 and 2020, CVD‐related mortality among individuals with diabetes has increased overall, with notable differences observed across age groups, gender, race/ethnicity, and geographic regions. This underscores the need for enhanced public health surveillance to gain a clearer understanding of CVD‐related mortality and to identify high‐risk demographic and regional subgroups for targeted interventions.

Our findings indicate that, following an initial decline, there has been a significant and concerning rise in mortality rates associated with cardiovascular disorders and DM. The Black or African American community is at the highest risk, with Oklahoma exhibiting the most alarming AAMRs in comparison to other regions. These results compel immediate action to raise awareness, strengthen multidisciplinary collaborations, and establish specialized clinics or programs to combat the escalating mortality rates in these high‐risk populations.

## Author Contributions

**Aiman Baloch:** conceptualization, methodology, software, data curation, investigation, formal analysis, visualization, project administration, writing – original draft, writing – review and editing. **Quang Dai La:** conceptualization, investigation, formal analysis, visualization, writing – original draft, writing – review and editing. **David M. Lo:** validation, supervision, writing – review and editing. **Jasneel Kahlam:** validation, supervision, writing – review and editing.

## Funding

The authors have nothing to report.

## Disclosure

The lead authors David M. Lo and Aiman Baloch affirm that this manuscript is an honest, accurate, and transparent account of the study being reported; that no important aspects of the study have been omitted; and that any discrepancies from the study as planned (and, if relevant, registered) have been explained.

## Conflicts of Interest

The authors declare no conflicts of interest.

## Supporting information

Supporting File

## Data Availability

The data that support the findings of this study are openly available in CDC WONDER at https://wonder.cdc.gov/ [27].
