# Supplementary material for: Trends in Age‐Adjusted Cardiovascular Mortality Rates Among the Diabetic Population of the United States: 1999–2020, CDC WONDER Retrospective Study
Source: Health Sci Rep. 2026 May 10;9(5):e72421. doi: 10.1002/hsr2.72421 (PMC13158147; doi:10.1002/hsr2.72421)
Supplement: Supplementary file 1 — Supporting File [file HSR2-9-e72421-s001.docx]

# Gender-Based Age-Adjusted Mortality Rates (AAMR) Joinpoint Regression Analysis

***1.1 MODEL ESTIMATES***

***1.1.1 MODEL STATISTICS***

| **Cohort** | **Number of Joinpoints** | **Number of Observations** | **Number of Parameters** | **Degrees of Freedom** | **Sum of Squared Errors** | **Mean Squared Error** | **Autocorrelation Parameter** |
| --- | --- | --- | --- | --- | --- | --- | --- |
| Male - 1 Joinpoint | 1 | 22 | 4 | 18 | 318.4159 | 17.6898 | Uncorrelated |
| Female - 1 Joinpoint | 1 | 22 | 4 | 18 | 276.7823 | 15.3768 | Uncorrelated |

*Table S1. Model Statistics for Gender-Based AAMR Trends (1999–2020)*

***1.1.2. ESTIMATED JOINPOINTS***

| **Cohort** | **Joinpoint** | **Estimate** | **Lower CI** | **Upper CI** |
| --- | --- | --- | --- | --- |
| Male - 1 Joinpoint | 1 | 2012 | 2003 | 2019 |
| Female - 1 Joinpoint | 1 | 2012 | 2003 | 2019 |

*Table S2. Estimated Joinpoints in Gender-Based AAMR Trends*

***1.2 MODEL SELECTION***

***1.2.1. MODEL SELECTION METHOD***

| **Cohort** | **Model Selection Method** |
| --- | --- |
| Male | Weighted BIC |
| Female | Weighted BIC |

*Table S3. Model Selection Method for Gender-Based Joinpoint Regression*

***1.2.2. TEST FOR NUMBER OF JOINPOINTS***

| **Cohort** | **Model** | **Number of Joinpoints** | **Number of Observations** | **Number of Parameters** | **Degrees of Freedom** | **Sum of Squared Errors** | **BIC** | **BIC3** | **Weight** | **WBIC~** |
| --- | --- | --- | --- | --- | --- | --- | --- | --- | --- | --- |
| Male | #1 | 0 Joinpoint(s) | 22 | 2 | 20 | 690.989 | 3.728085 | 3.728085 | - | 3.728085 |
| Male | #2 | 1 Joinpoint(s) ^ | 22 | 4 | 18 | 318.4159 | 3.234324 | 3.374826 | 0.539188 | 3.310081 |
| Male | #3 | 2 Joinpoint(s) | 22 | 6 | 16 | 296.5704 | 3.444254 | 3.725258 | 0.506667 | 3.586629 |
| Male | #4 | 3 Joinpoint(s) | 22 | 8 | 14 | 286.3928 | 3.690337 | 4.111843 | 0.798658 | 4.026976 |
| Male | #5 | 4 Joinpoint(s) | 22 | 10 | 12 | 278.4312 | 3.943148 | 4.505156 | 0.895246 | 4.446283 |
| Female | #1 | 0 Joinpoint(s) | 22 | 2 | 20 | 550.5491 | 3.500878 | 3.500878 | - | 3.500878 |
| Female | #2 | 1 Joinpoint(s) ^ | 22 | 4 | 18 | 276.7823 | 3.094196 | 3.234698 | 0.497262 | 3.164063 |
| Female | #3 | 2 Joinpoint(s) | 22 | 6 | 16 | 235.7963 | 3.214937 | 3.495941 | 0.783837 | 3.435199 |
| Female | #4 | 3 Joinpoint(s) | 22 | 8 | 14 | 222.2285 | 3.436679 | 3.858185 | 0.95856 | 3.840718 |
| Female | #5 | 4 Joinpoint(s) | 22 | 10 | 12 | 214.0317 | 3.680101 | 4.242109 | 0.95856 | 4.218819 |

*Table S4. Permutation Test for Number of Joinpoints*

# Racial Based Age-Adjusted Mortality Rates (AAMR) Joinpoint Regression Analysis

***2.1 MODEL ESTIMALES***

***2.1.1. MODEL STATISTICS***

| **Cohort** | **Number of Joinpoints** | **Number of Observations** | **Number of Parameters** | **Degrees of Freedom** | **Sum of Squared Errors** | **Mean Squared Error** | **Autocorrelation Parameter** |
| --- | --- | --- | --- | --- | --- | --- | --- |
| White - 2 Joinpoints | 2 | 22 | 6 | 16 | 268.7259 | 16.7954 | Uncorrelated |
| Asian or Pacific Islander - 2 Joinpoints | 2 | 22 | 6 | 16 | 41.9929 | 2.6246 | Uncorrelated |
| Black or African American - 1 Joinpoint | 1 | 22 | 4 | 18 | 399.7043 | 22.2058 | Uncorrelated |
| American Indian or Alaska Native - 1 Joinpoint | 1 | 22 | 4 | 18 | 17.5785 | 0.9766 | Uncorrelated |
| Hispanic or Latino - 1 Joinpoint | 1 | 22 | 4 | 18 | 40.3653 | 2.2425 | Uncorrelated |

*Table S5. Model Statistics for Racial-Based AAMR Trends (1999–2020)*

***2.1.2. ESTIMATED JOINPOINTS***

| **Cohort** | **Joinpoint** | **Estimate** | **Lower CI** | **Upper CI** |
| --- | --- | --- | --- | --- |
| White - 2 Joinpoints | 1 | 2012 | 2002 | 2014 |
| White - 2 Joinpoints | 2 | 2018 | 2014 | 2018 |
| Asian or Pacific Islander - 2 Joinpoints | 1 | 2013 | 2001 | 2015 |
| Asian or Pacific Islander - 2 Joinpoints | 2 | 2018 | 2014 | 2018 |
| Black or African American - 1 Joinpoint | 1 | 2018 | 2016 | 2018 |
| American Indian or Alaska Native - 1 Joinpoint | 1 | 2011 | 2008 | 2014 |
| Hispanic or Latino - 1 Joinpoint | 1 | 2016 | 2004 | 2018 |

*Table S6. Estimated Joinpoints in Racial-Based AAMR Trends*

***2.2. MODEL SELECTION***

***2.2.1. MODEL SELECTION METHOD***

| **Cohort** | **Model Selection Method** |
| --- | --- |
| White | Weighted BIC |
| Asian or Pacific Islander | Weighted BIC |
| Black or African American | Weighted BIC |
| American Indian or Alaska Native | Weighted BIC |
| Hispanic or Latino | Weighted BIC |

*Table S7. Model Selection Method for Racial-Based Joinpoint Regression*

***2.2.2. TEST FOR NUMBER OF JOINTPOINTS***

| **Cohort** | **Model** | **Number of Joinpoints** | **Number of Observations** | **Number of Parameters** | **Degrees of Freedom** | **Sum of Squared Errors** | **BIC** | **BIC3** |
| --- | --- | --- | --- | --- | --- | --- | --- | --- |
| White | #1 | 0 Joinpoint(s) | 22 | 2 | 20 | 3660.1011812 | 5.3952075 | 5.3952075 |
| White | #2 | 1 Joinpoint(s) | 22 | 4 | 18 | 498.2361918 | 3.6820395 | 3.8225414 |
| White | #3 | 2 Joinpoint(s) ^ | 22 | 6 | 16 | 268.7259277 | 3.3456611 | 3.6266650 |
| White | #4 | 3 Joinpoint(s) | 22 | 8 | 14 | 233.2692871 | 3.4851665 | 3.9066723 |
| White | #5 | 4 Joinpoint(s) | 22 | 10 | 12 | 195.4089521 | 3.5890714 | 4.1510791 |
| Asian or Pacific Islander | #1 | 0 Joinpoint(s) | 22 | 2 | 20 | 362.3661188 | 3.0826165 | 3.0826165 |
| Asian or Pacific Islander | #2 | 1 Joinpoint(s) | 22 | 4 | 18 | 68.9862777 | 1.7048729 | 1.8453748 |
| Asian or Pacific Islander | #3 | 2 Joinpoint(s) ^ | 22 | 6 | 16 | 41.9928849 | 1.4894693 | 1.7704732 |
| Asian or Pacific Islander | #4 | 3 Joinpoint(s) | 22 | 8 | 14 | 39.1939502 | 1.7014954 | 2.1230012 |
| Asian or Pacific Islander | #5 | 4 Joinpoint(s) | 22 | 10 | 12 | 37.7271951 | 1.9443580 | 2.5063658 |
| Black or African American | #1 | 0 Joinpoint(s) | 22 | 2 | 20 | 1626.3662140 | 4.5840649 | 4.5840649 |
| Black or African American | #2 | 1 Joinpoint(s) ^ | 22 | 4 | 18 | 399.7043094 | 3.4616903 | 3.6021922 |
| Black or African American | #3 | 2 Joinpoint(s) | 22 | 6 | 16 | 327.2323649 | 3.5426396 | 3.8236435 |
| Black or African American | #4 | 3 Joinpoint(s) | 22 | 8 | 14 | 176.2972636 | 3.2051446 | 3.6266503 |
| Black or African American | #5 | 4 Joinpoint(s) | 22 | 10 | 12 | 173.6536699 | 3.4710398 | 4.0330475 |
| American Indian or Alaska Native | #1 | 0 Joinpoint(s) | 22 | 2 | 20 | 54.4197415 | 1.1866884 | 1.1866884 |
| American Indian or Alaska Native | #2 | 1 Joinpoint(s) ^ | 22 | 4 | 18 | 17.5784738 | 0.3376403 | 0.4781423 |
| American Indian or Alaska Native | #3 | 2 Joinpoint(s) | 22 | 6 | 16 | 15.3993649 | 0.4862954 | 0.7672992 |
| American Indian or Alaska Native | #4 | 3 Joinpoint(s) | 22 | 8 | 14 | 14.3702214 | 0.6981311 | 1.1196369 |
| American Indian or Alaska Native | #5 | 4 Joinpoint(s) | 22 | 10 | 12 | 13.8322972 | 0.9409831 | 1.5029908 |
| Hispanic or Latino | #1 | 0 Joinpoint(s) | 22 | 2 | 20 | 71.4419606 | 1.4588468 | 1.4588468 |
| Hispanic or Latino | #2 | 1 Joinpoint(s) ^ | 22 | 4 | 18 | 40.3652886 | 1.1689355 | 1.3094374 |
| Hispanic or Latino | #3 | 2 Joinpoint(s) | 22 | 6 | 16 | 36.1085185 | 1.3384979 | 1.6195018 |
| Hispanic or Latino | #4 | 3 Joinpoint(s) | 22 | 8 | 14 | 31.8145608 | 1.4928971 | 1.9144028 |
| Hispanic or Latino | #5 | 4 Joinpoint(s) | 22 | 10 | 12 | 29.3381161 | 1.6928644 | 2.2548721 |

*Table S8. Permutation Test for Number of Joinpoints*
